# Fast and Efficient Differentiation of Mouse Embryonic Stem Cells Into ATP-Responsive Astrocytes

**DOI:** 10.3389/fncel.2019.00579

**Published:** 2020-01-21

**Authors:** Deppo S. Juneja, Slawomir Nasuto, Evangelos Delivopoulos

**Affiliations:** School of Biological Sciences, University of Reading, Reading, United Kingdom

**Keywords:** stem cell, purinergic, adenosine triphosphate, GFAP, S100β, astrocyte

## Abstract

Astrocytes are multifunctional cells in the CNS, involved in the regulation of neurovascular coupling, the modulation of electrolytes, and the cycling of neurotransmitters at synapses. Induction of astrocytes from stem cells remains a largely underdeveloped area, as current protocols are time consuming, lack granularity in astrocytic subtype generation, and often are not as efficient as neural induction methods. In this paper we present an efficient method to differentiate astrocytes from mouse embryonic stem cells. Our technique uses a cell suspension protocol to produce embryoid bodies (EBs) that are neurally inducted and seeded onto laminin coated surfaces. Plated EBs attach to the surface and release migrating cells to their surrounding environment, which are further inducted into the astrocytic lineage, through an optimized, heparin-based media. Characterization and functional assessment of the cells consists of immunofluorescent labeling for specific astrocytic proteins and sensitivity to adenosine triphosphate (ATP) stimulation. Our experimental results show that even at the earliest stages of the protocol, cells are positive for astrocytic markers (GFAP, ALDH1L1, S100β, and GLAST) with variant expression patterns and purinergic receptors (P2Y). Generated astrocytes also exhibit differential Ca^2+^ transients upon stimulation with ATP, which evolve over the differentiation period. Metabotropic purinoceptors P2Y_1_R are expressed and we offer preliminary evidence that metabotropic purinoceptors contribute to Ca^2+^ transients. Our protocol is simple, efficient and fast, facilitating its use in multiple investigations, particularly *in vitro* studies of engineered neural networks.

## Introduction

Astrocytes are present in the nervous system in approximately equal numbers to neurons and have multifunctional physical connections with synapses, other astrocytes and blood vessels (Lent et al., 2012). In the last 20 years, numerous investigations have revealed associations between neurological disorders and astrocytic malfunction. For example, loss of dopaminergic neurons in Parkinson’s disease is linked to abnormal accumulation of α-synuclein by both neurons and astrocytes (Lee et al., 2010). In a Huntington’s disease mouse model, an astrocytic ion channel deficit (Kir4.1) was revealed to be both the cause of medium spiny neuron dysfunction and a therapeutic target (Tong et al., 2016). As a result of such findings and the recent demonstration of transplanted astrocyte reprograming into functional neurons (Zhang L. et al., 2015), astrocytes are now seen as principal candidates for transplantation studies and cell replacement therapy (Zhang Y. et al., 2016). It is now accepted, that astrocytes participate in information processing in the brain, even enhancing synaptic plasticity and learning in cross species transplantations (Han et al., 2013). Therefore, the efficient derivation of functional astrocytes from differentiating stem cells is rapidly becoming the focus of many research labs, as it is seen as a crucial step in understanding brain function in health and disease. Furthermore, from a neuroengineering perspective, facilitation of astrocyte procurement enables multiple studies, investigating neural circuit patterning (Delivopoulos et al., 2009; Delivopoulos and Murray, 2011; Unsworth et al., 2011; Raos et al., 2018), and development (Clarke and Barres, 2013) both *in vitro* and *in vivo*.

Differentiation of astrocytes from pluripotent stem cells has gained considerable momentum in the past decade. Proposed methods use embryoid bodies (EBs) of human induced pluripotent stem cells (hIPSCs) to generate glial progenitor cells (GPC) (Santos et al., 2017), neural progenitor cells (NPC) (Tcw et al., 2017), or neuroepithelial cells (Krencik et al., 2011), which are then induced into human astrocytes. Murine astrocytes have been generated from reprogramed fibroblasts via small molecules (Tian et al., 2016) and also from NPC with the use of BMP4 (Kleiderman et al., 2016). A two-step differentiation scheme was employed by Kuegler et al. (2012) to derive functional murine astrocytes from the CGR8 mouse embryonic stem cell line (mESC). Recently, Tiwari et al. (2018) illustrated that ATF3 and NFIA transcription factors drive astrocyte differentiation from mESC, while RUNTX2 promotes astrocyte maturation. Nonetheless, the majority of current protocols require a considerable investment of time, before producing functional astrocytes that express expected markers. For example, a protocol by Krencik et al. that was based on basic fibroblast growth factor (FGF2) and epidermal growth factor (EGF) required 6 months of differentiation (Krencik and Zhang, 2011). Emdad et al. (2011) shortened astrocyte differentiation to 80 days, in a protocol requiring manual selection of rosettes on the initial stage of differentiation (Emdad et al., 2011). Roybon et al. (2013) generated both reactive and quiescent astrocytes in a similar time frame (90 days) (Roybon et al., 2013), while Zhou et al. (2016) presented an accelerated method for astrocyte differentiation (28 days), based on floating neurospheres.

In glial biology, astrocytes were traditionally considered a homogenous population of cells. This misconception was later extended to astrocytes differentiated from stem cells. However, recent studies have allowed a greater appreciation of the diverse regional profiles and functional roles of primary astrocytes. For example, functional heterogeneity of astrocytes within the glial scar may have important implications during gliosis and CNS regeneration (Adams and Gallo, 2018). This has been linked to variant isoforms of GFAP (α, β, γ, δ, and κ), which are expressed heterogeneously in both the healthy and pathological CNS (Moeton et al., 2016; van Bodegraven et al., 2019). Equally, region specific responses of astrocytes to neurotrophins (NGF, BDNF) affect wound closure at lesion sites (Cragnolini et al., 2018), while cellular and regional diversity of astrocytes is related to differential transcriptomic changes, during multiple sclerosis and potentially other neurodegenerative diseases (Itoh et al., 2018). A possible basis for astrocyte heterogeneity may be the spatiotemporal patterns of morphogens that emerge during development of the mammalian CNS and diversify both glial and neuronal progenitors (Ben Haim and Rowitch, 2017). Regardless of origin, astrocyte diversity in gene expression, morphology and function has been established, and appears essential in both physiological and pathological conditions.

In this study, we derive functional astrocytes from mouse embryonic stem cells, after only 13 days of differentiation. To our knowledge, our protocol is currently the fastest (<2 weeks), reproducible method to generate astrocytes from mESC. Our technique employs a cell suspension protocol proposed by Peljto et al. (2010) to produce EBs, which are neuralized via retinoic acid treatment for 6 days. EBs are then plated on laminin coated surfaces and cells resembling astrocytes start migrating away from the seeded EBs, within 2 days *in vitro*. We characterized migrating cells with different techniques at 7, 14, 21, and 28 DIV and our results demonstrate that they express established astrocytic markers (GFAP, ALDH1L1, S100β, and GLAST) associated to astrocytic development and glutamate uptake. We offer evidence that the produced population of astrocytes is heterogeneous, with cells exhibiting different markers and transient Ca^2+^ responses upon adenosine triphosphate (ATP) stimulation. These responses to ATP could be due to metabotropic purinoceptor (P2Y_1_R) activation and the downstream release of intracellular Ca^2+^ from stores.

## Materials and Methods

### Maintenance of Mouse Embryonic Stem Cell Line

The mouse embryonic stem cell (mESC) line CGR8 (derived from Mus musculus, strain 129) were incubated at 37°C, 5% CO2, in gelatine coated flasks, in a media composed of DMEM, supplemented with 10% fetal calf serum (FCS) (Gibco Industries, Inc., Langley, OK, United States), 1% penicillin/streptomycin Pen/Strep), 1% L-glutamine (Life Technologies, Paisley, United Kingdom), 100 μM 2- Mercaptoethanol and LIF (Leukemia Inhibitory Factor) (1000 units/mL) (Sigma Aldrich, United Kingdom). Cells were passaged every 2 days, and assessed daily for confluence.

### Differentiation of Mouse Embryonic Stem Cell Line Into Astrocytes

**FIGURE 1:**
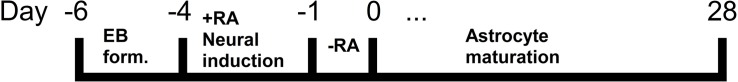
Schematic representation of the protocol used for the differentiation of mouse embryonic stem cell lines (mESCs) into astrocytes over a total time of 2–5 weeks. Mouse ESCs were used to generate embryoid bodies (EBs), which were seeded onto laminated glass coverslips. Migrating astrocytes were detected and labeled from DIV 7 to DIV 28.

Maintenance cultures were used for differentiation at high confluence (60–80%). The process of differentiation followed a combination and adjustment of two different protocols from Peljto et al. (2010) and Kuegler et al. (2012) as shown in Figure 1. Briefly, 500,000 cells/ml were seeded onto low attachment petri dishes, and suspended in 10 ml of ADFNK media (Advanced DMEM/F-12:Neurobasal medium (1:1) supplemented with 10% Knockout Serum Replacement, 1% L- Glutamine, 100 μM β-mercaptoethanol, and 1% Pen/Strep), devoid of LIF. After 2 days, single cells formed EBs. EB were exposed to fresh ADFNK media containing 1 μM retinoic acid (RA) (Sigma Aldrich, United Kingdom) at day 2. At day 5, RA was removed and fresh ADFNK media was added. At day 6 an altered version of Kuegler et al. (2012) protocol was utilized. Twenty to fifty intact EB were seeded directly onto laminin coated 24 well plates containing astrocyte differentiation media (ADMEM/F12, 2% FBS, N2, 1% L-glutamine, 1% Pen/Strep, 100 μM β-mercaptoethanol and 50 μg/mL heparin (Robertson and Goldstein, 1988; Nagayasu et al., 2005) (Sigma Aldrich, United Kingdom). The cells were assessed via bright field microscopy every day and cultured for up to 28 days. The media was changed every 2 days.

### Immunofluorescence Labeling

Cells were washed with PBS, at 7, 14, 21, and 28 DIV, fixed with 3.7% formaldehyde in PBS for 30 minutes, and permeabilized with 0.02% Triton X-100 diluted in 10% goat serum for 15 min. Subsequently, cells were washed with PBS and 10% goat serum was added to block against non-specific binding for 2 h. Cells were then stained with appropriate primary and secondary antibodies (see Supplementary Table S1). Nuclei were counterstained with H-33342 (Hoechst dye). Primary and secondary antibodies were left on the fixed cultures for 2 h at room temperature, or overnight at 4°C.

### Image Acquisition and Analysis, Quantification of Marker Positive Cells, and Co-localization

Images were acquired using an Axio Imager microscope (Zeiss, Germany) and a Nikon A1-R confocal microscope with a resonant scanner. Separate digital images (red 594 nm, green 488 nm, and blue 350 nm) of astrocyte stains and DNA (Hoechst 33342) were captured with a × 20 objective (1040 × 1388 pixels) from 3 non-overlapping microscopic fields per glass coverslip (AxioCam CCD digital camera (Carl Zeiss). Focus was adjusted for each new field, but fluorescent illumination and exposure were kept constant to improve consistency during analysis. Images were also collected with ×5 and ×10 objectives, from wider fields of view. Scale bars were calibrated using AxioVision software (release 4.6.3, Carl Zeiss).

Image analysis was performed in ImageJ (NIH – National Institutes of Health) and MATLAB. For analysis of cell body and nuclei, images were split into individual channels from RGB (red 594 nm and green 488 nm, blue 350 nm) and thresholded utilizing the Otsu method (Vala and Baxi, 2013) to remove any uneven illumination. Any debris and unwanted artifacts were cropped from the image. The original single channel image was overlaid on top of the thresholded image and Pearson’s correlation coefficient (PCC) analysis was performed to assess their similarity. If PCC < 0.8 the image was thresholded again, otherwise the image was analyzed for number of marker positive cells and percentage area covered by marker positive cells. Co-localization analysis was performed on co-labeled cultures, using Pearson correlation coefficient analysis derived from the following equation (see Adler and Parmryd, 2010) for detailed methodology):

$$r=\frac{\sum_{i}\left(R\,𝔦-\tilde{R}\right).\left(G\,i-\tilde{G}\right)}{\sqrt{\sum_{i}{\left(R\,𝔦-\tilde{R}\right)}^{2}.\sum_{i}{\left(G\,i-\tilde{G}\right)}^{2}}},$$

where $\tilde{R}$ and $\tilde{G}$ are the average intensities in the red and green channels, respectively. Perfect positive (PCC = 1) and negative (PCC = −1) correlations denote that the two markers are expressed by either an individual or distinct cell populations, respectively. A PCC of zero suggests the absence of any relationship between the expressions of the two markers.

### Calcium Spectrofluorometry

Astrocyte [Ca^2+^]_*i*_ concentration dynamics were evaluated using Ca^2+^ sensitive fluorescent dye Fluo-4/AM (Molecular Probes). Cells were stimulated pharmacologically with ATP (Sigma Aldrich, Poole, United Kingdom) at 50 uM concentration. Prior to recording, cells loaded with Fluo-4/AM (2.5 μM) for 30 min at 37°C, 5% CO2 (Molecular Probes) (Neary et al., 1999; Gee et al., 2000; James and Butt, 2001). Subsequently, cells were thoroughly rinsed three times, with Hank’s Balanced Salt Solution (HBSS) to remove extracellular traces of the dye and to complete de-esterification. In separate experiments 100 μM Suramin/10 μM MRS2179 was applied for 30 min before imaging. In one culture, 10 μM of phospholipase C (PLC) inhibitor U73122 was applied for 30 min prior to imaging. All compounds were rinsed thoroughly three times with HBSS before imaging. Excitation and emission wavelengths were 494 nm and 516 nm, respectively. All fluorescence measurements were made at 37°C (Warner Instruments). Changes in [Ca^2+^]_*i*_ were detected with an inverted Nikon Eclipse TE2000-S microscope (Nikon) equipped with a xenon arc lamp (Sutter Instruments).

### Calcium Spectrofluorometry Analysis

All data analysis was performed offline and data assessed with Clampfit 10, WinFlour software packages (Strathclyde University) and modified MATLAB algorithms. Astrocytes were identified at 488 nm excitation, and cell bodies within a single plane of focus were selected as regions of interest (ROI). Five to ten ROI were simultaneously recorded from each glass coverslip in each experiment. Three separate experiments were performed. Fluorescence intensity was normalized by dividing fluorescence at time t by the mean intensity between 0–20 s, before addition of agonist (F/F0). Waveforms were filtered using a low pass filter (Otsu et al., 2015) and the following parameters characterizing them were estimated: rise time (RT) (time taken from 10% to 90% of peak amplitude), decay time constant tau (exponential curve (*f* (*t*) = *a*^∗^exp(−*t/tau*)) was fit from peak amplitude till the end of the experiment) and latency (time taken from ATP application to initiation of a calcium transient) (Kang and Othmer, 2009; Okada et al., 2012; Hashioka et al., 2014). Waveforms were categorized as either monophasic or biphasic, depending on whether the decay phase exhibited a sustained response after the half-amplitude of peak response. The end of the experiment was taken at 300 s or where the error of the fitted exponential curve *f* (*t*) (see decay tau above) to the actual waveform was equal or greater than 10% (if this occurred earlier).

### Statistical Analysis

Experiments with immunofluorescent labeling and Ca^2+^ imaging were performed in triplicates. In each of the three experiments 3 glass coverslips for each timepoint (7, 14, 21, and 28 DIV) were cultured and fixed for a total of 9 glass coverslips. Three images were collected from each glass coverslip (27 images were analyzed per timepoint per marker across all three experiments). For Ca^2+^ imaging 3 glass coverslips were recorded from 3 separate experiments. During analysis, waveforms from 5 to 10 ROI (cell somata) were decomposed as described above.

Data are presented as means ± SEM and the statistical differences were tested by ANOVA with Bonferroni’s Multiple Comparison *post hoc* or Mann-Whitney tests where appropriate, using GraphPad Prism 6.0 (Graphpad Software, La Jolla, CA, United States). Statistical significance was assumed when *P* < 0.05, and was indicated by an asterisk on the respective data point in the figure. Two asterisks indicated *P* < 0.01 and three *P* < 0.001.

## Results

### Immunocytochemical Analysis of Mouse Embryonic Stem Cell Line Derived Astrocytes

**FIGURE 2:**
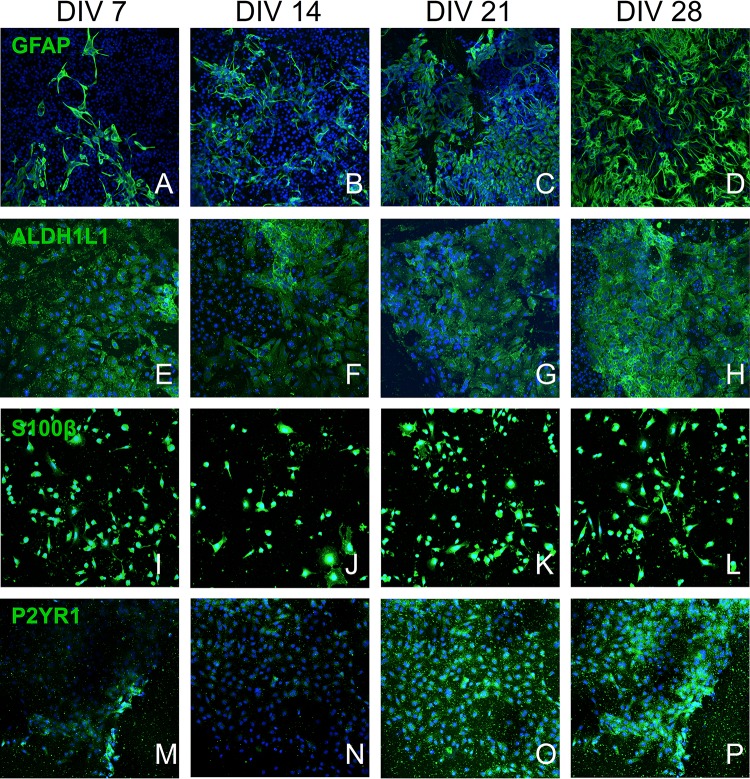
Confocal culture micrographs (7–28 DIV) of astrocytes inducted from mESC, immunofluorescently labeled for established astrocytic markers: **(A–D)** – GFAP, **(E–H)** – ALDH1L1, **(I–L)** – S100B, and **(M–P)** – P2Y_1_R. Cell nuclei marker H-33342 (blue stain). Scale bar 20 μm.

The mESC induction and differentiation protocols generated a heterogeneous population of astrocytes. The EBs formed during the neural induction process were plated onto laminin coated coverslips (10–20 EBs per coverslip) and were cultured in astrocyte medium. Plated EBs attached to the surface and projected cells to their surrounding environment. We used a broad panel of fluorescent probes to astrocytic markers to characterize the differentiating mESC at DIV 7, 14, 21, and 28. The following astrocytic and purinoreceptor markers were used: GFAP (Figures 2A–D; Eng et al., 2000), ALDH1L1 (Figures 2E–H; Cahoy et al., 2008), S100β (Figures 2I–L; Raponi et al., 2007), and P2Y_1_R (Figures 2M–P; Hamilton et al., 2008).

**FIGURE 3:**
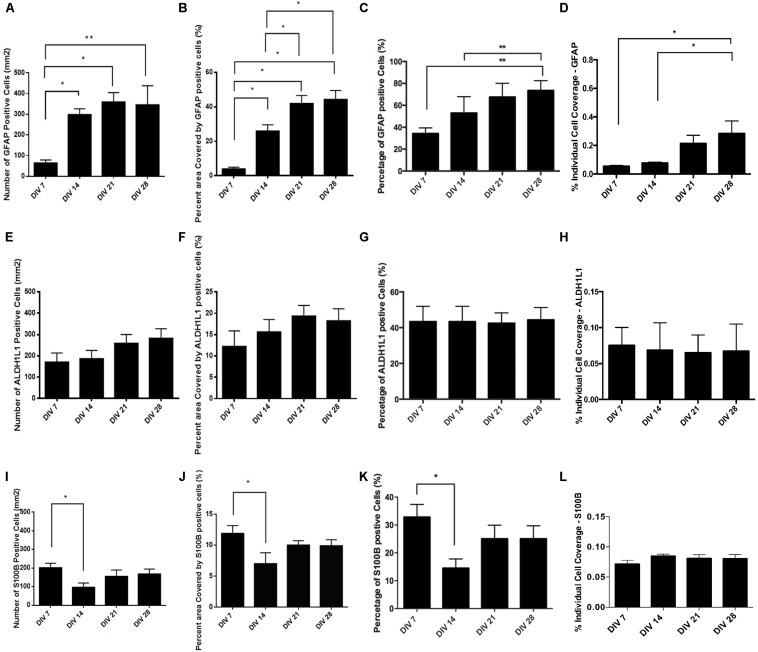
Quantification of astrocyte marker expression: **(A–D)** – GFAP, **(E–H)** – ALDH1L1, **(I–L)** – S100B. **(A,E,I)** absolute number of marker positive cells per mm^2^. **(B,F,J)** Percent (%) area covered by marker positive cells. **(C,G,K)** Percent (%) of marker positive cells. **(D,H,L)** Percent of area covered by an average marker positive cell. Data expressed as mean ± SEM from three separate experiments, ^∗^*P* < 0.05 and ^∗∗^*P* < 0.01.

The number of GFAP^+^ cells increased over four weeks (Figure 3A) and at DIV 14 approximately half of the cell population was GFAP^+^ (Figure 3C: 52.93% ± 14.94%, *n* = 9) and exhibited typical astrocytic morphology (Figure 2B). This percentage significantly increased during the following weeks and plateaued at DIV 28 (Figure 3C: 73.68% ± 9.03%, *n* = 9). Assessment of the relative surface area covered by GFAP^+^ cells revealed a statistically significant increase (*p* < 0.05) from less than 5% surface coverage at DIV7 (Figure 3B: 3.88% ± 1.060%, *n* = 9) to more than 40% at DIV28 (Figure 3B: 44.23% ± 5.268%, *n* = 9). We also calculated the area covered by the average individual GFAP^+^ cell at different DIV (Figure 3D). We found a significant (*p* < 0.05) doubling in GFAP^+^ cell size from DIV 7 (Figure 3D: 0.054% ± 0.0057%, *n* = 9) to DIV28 (Figure 3D: 0.284% ± 0.088%, *n* = 9), which coincided with a shift from a “dinner plate” (Molofsky and Deneen, 2015) morphology at DIV 7 (Figure 2A) to an enlarged, complex morphology with elongated processes (typically seen in primary astrocytes) at DIV 28 (Figure 2D).

We observed that the number and percentage of S100β^+^ cells dropped significantly (*P* < 0.05) between DIV 7 (Figures 3I,K: 32.82% ± 4.61%, *n* = 9) and DIV 14 (Figure 3K: 14.52% ± 3.29%, *n* = 9). The following 2 weeks S100β^+^ cells recovered and plateaued at DIV 28 (Figure 3K: 25.04% ± 4.67%, *n* = 9). During neural progenitor differentiation S100β is repressed and reappears in mature astrocytic phenotypes. Its role also changes from regulating GFAP conformation and cell proliferation to binding calcium, and mediating inflammatory markers (Selinfreund et al., 1991; Raponi et al., 2007). The relative surface area covered by S100β^+^ cells was highest at DIV 7 (Figure 3J: 13.56% ± 1.55%, *n* = 9), with a statistically significant reduction (*p* < 0.05) at DIV 14 (Figure 3J: 7.01% ± 1.78%, *n* = 9), which coincided with the decrease in the percentage of S100β^+^ cells. At DIV 21 and 28, cell coverage followed a similar trend to the percentage of S100β^+^ cells. Analysis of area coverage of an average individual S100β^+^ cell showed no significant differences across DIVs and was stable at approximately 0.08% (Figure 3L).

Analysis of ALDH1L1 immunolabeled cultures revealed no significant difference in the number or percentage of ALDH1L1^+^ cells from DIV 7 to DIV 28 (Figures 3E,G: stable at approximately 40%). A non-significant upward trend was observable on the relative surface area coverage (Figure 3F) from DIV 7 to DIV 28, whereas relative area coverage of an average ALDH1L1^+^ cell remained stable at approximately 0.07% (Figure 3H).

Pluripotency gene Nanog Homebox (*Nanog*) was minimally expressed (see Supplementary Material) post DIV 7, indicating the limited presence of stem cells amongst the generated astrocyte population (Supplementary Figure S1A). We did not detect any expression of the allograft inflammatory factor 1 (*iba1* present in microglia) in our PCR screening (unpublished data) at DIV 7 or later. We also stained but did not detect any expression of NESTIN at DIV 7 or later (Supplementary Figure S1B), suggesting absence or very limited presence of neural progenitors, during the EB seeding/astrocyte induction phase of our differentiation protocol.

### Co-localization of Astrocytic Markers

**FIGURE 4:**
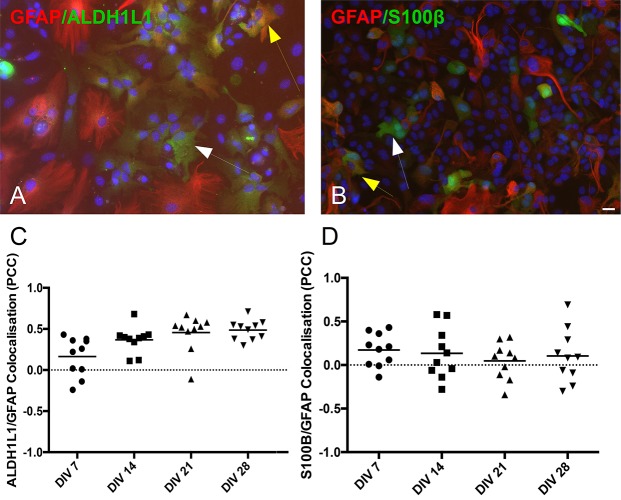
Co-localization of astrocytic markers (GFAP, ALDH1L1, and S100β). **(A)** Culture example at DIV 21 of astrocytes co-expressing GFAP and ALDH1L1 (yellow arrow) or expressing only ALDH1L1 (white arrow). **(B)** Culture example at DIV 21 of astrocytes co-expressing GFAP and S100β (yellow arrow) or expressing only S100β (white arrow). Cell nuclei marker H-33342 (blue stain). Scale bar 20 μm. **(C,D)** Analysis of co-localization across different days *in vitro* reveals heterogeneity of differentiated astrocytes in protein expression. Pearson’s correlation coefficient of GFAP/ALDH1L1 and GFAP/S100β stains from DIV 7 to DIV 28.

Astrocytes generated *in vitro* are not as homogenous in marker expression as previously assumed. To highlight the existence of subpopulations of astrocytes, we performed double-immunolabeling of GFAP with either ALDH1L1 (Figure 4A) or S100β (Figure 4B). In the two figures the white arrows point to cells expressing only one protein, ALDH1L1 or S100β, while the yellow arrows point to cells expressing both proteins. These culture micrographs from DIV 21 highlight the presence of astrocytes at different developmental stages, as GFAP is usually expressed in more mature astrocytes. We calculated the PCC of the two stains and revealed that not all cells express all astrocytic markers simultaneously, illustrating the existence of astrocytic sub-populations. Analysis of ALDH1L1 and GFAP co-staining revealed a positive correlation, which was increasing toward the late time-points (Figure 4C PCC at DIV 7 0.165 ± 0.07 *n* = 9 and DIV 28 0.486 ± 0.04 *n* = 9). S100β and GFAP stains were not correlated throughout the culture, although there were multiple cells expressing only one or both of the markers (Figure 4D PCC at DIV7 0.173 ± 0.06 *n* = 9 and DIV 28 0.104 ± 0.09 *n* = 9).

**FIGURE 5:**
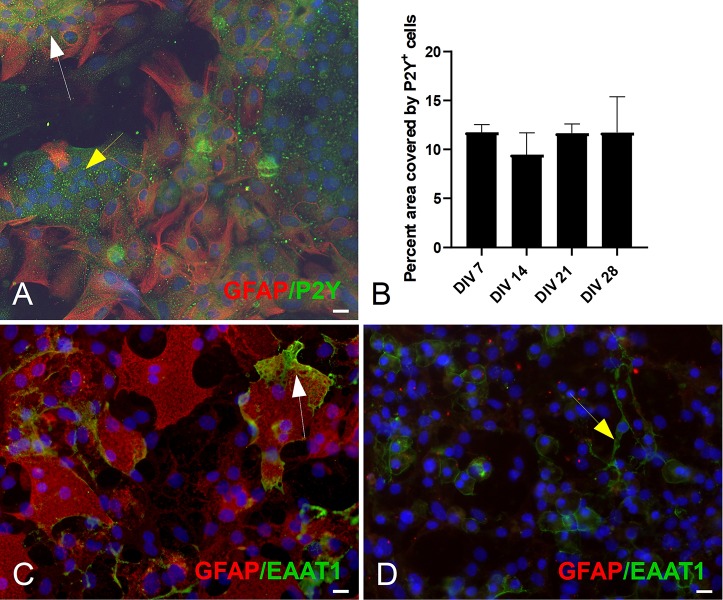
Double ICC staining of mESC derived astrocytes for markers GFAP, P2Y_1_R. **(A)** Culture example at DIV 21 of GFAP^+^ (white arrow) astrocytes and GFAP^–^ cells (yellow arrow) expressing the metabotropic P2Y_1_R receptor. **(B)** Quantification of P2Y_1_R expression: percent (%) area covered by P2Y_1_R^+^ cells. **(C)** Culture example at DIV 21 of GFAP^+^ (white arrow) expressing the Glutamate Aspartate Transporter (GLAST, also known as EAAT1). **(D)** Culture example at DIV 21 of GFAP^–^ (yellow arrow) expressing GLAST. Cell nuclei marker H-33342 (blue stain). Scale bars 20 μm.

The expression of purinergic receptors was not uniform across all GFAP^+^ astrocytes. P2Y_1_R purinoceptors expression was evident in both GFAP^+^ and GFAP^–^ populations (Figure 5A, white and yellow arrows, respectively). The percent area covered by P2Y_1_R^+^ did not change significantly across different DIV (Figure 5B).

The glutamate-aspartate transporter (GLAST or EAAT1) was also detected and was expressed predominantly in GFAP^+^ astrocytes (Figure 5C, white arrow). However, we did see examples of EAAT1^+^/GFAP^–^ cells (Figure 5D, yellow arrow).

### Mouse Embryonic Stem Cell Line-Derived Astrocytes Exhibit Ca^2+^ Transients Upon Adenosine Triphosphate Stimulation

**FIGURE 6:**
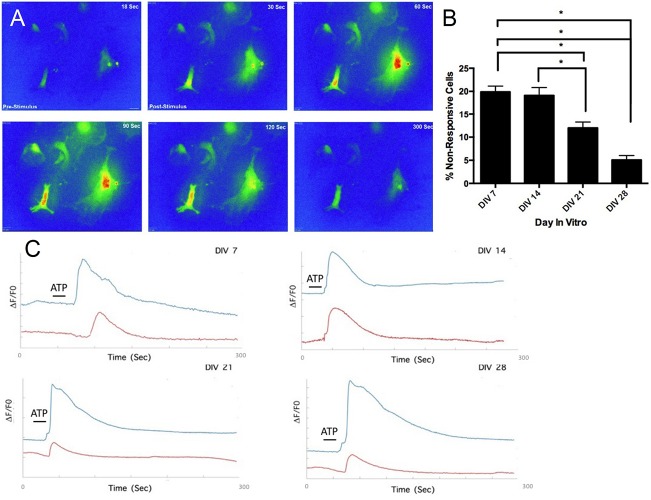
Functional assessment of mESC derived astrocytes. **(A)** Time-lapse heat-map of activated astrocytes after stimulation **(A)** via adenosine triphosphate (ATP) (50 μM). **(B)** Decrease in percentage of non-responsive cells after ATP stimulation, over the course of 4 weeks. **(C)** DIV 7-DIV 28 astrocytes treated with suramin exhibit stunted Ca^2+^ responses after ATP application (red traces), compared to untreated astrocytes (blue traces), ^∗^*P* < 0.05.

We examined whether the generated astrocytes had functionally mature and responsive purinergic pathways (Figure 6A). Stimulation of astrocytes via ATP (50 μM) generated slow wave-like Ca^2+^ transients, a hallmark of astrocyte function (Bazargani and Attwell, 2016; Shigetomi et al., 2016). A time-lapse sequence of a 5 minute recording is illustrated in Figure 6A, where calcium concentration in multiple regions of interest (e.g., middle right and middle left) increases (from 30 to 60 s) and returns to initial levels (from 90 to 300 s). Quantification of the percentage of astrocytes displaying transients showed a significant (*p* < 0.05) decrease in percentage of non-responsive cells from approximately 20% at DIV 7 to approximately 5% at DIV 28 (Figure 6B). Cellular responses to ATP stimulus were inhibited by the broad-spectrum P2X/2Y antagonist suramin, which blocks metabotropic (P2Y_1_R) and ionotropic (P2X_7_) purinoceptors (Bernstein et al., 1998). Upon ATP stimulation, we observed a stunted response in suramin treated cells (Figure 6C) across all time-points, providing further validation that prior Ca^2+^ responses were indeed purinergic.

**FIGURE 7:**
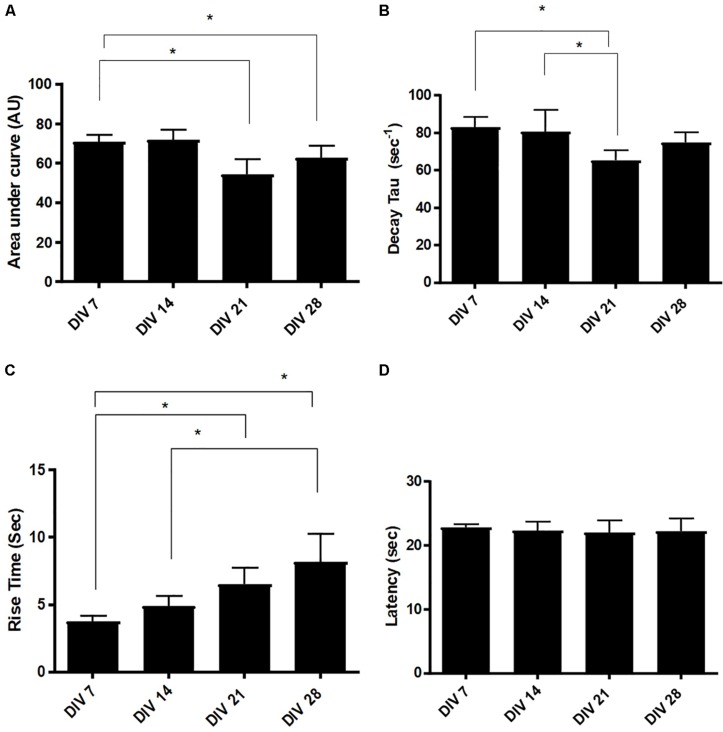
Evolution of Ca^2+^ transient parameters in mESC derived astrocytes. **(A)** Area under the curve (AUC), **(B)** decay tau (DT), **(C)** rise time (RT), and **(D)** latency. Statistical analysis reveals a significant increase in response RT from DIV7/14 to DIV 21 and DIV 28. Latency remains stable across different days *in vitro* (AU, arbitrary units, data expressed as mean ± SEM from three separate experiments, *n* = 30 astrocytes, ^∗^*P* < 0.05).

We decomposed the Ca^2+^ waveforms into three different components: rise time (RT), area under the curve (AUC) and decay tau (DT) (James et al., 2011). We also calculated the latency between the application of ATP and the rise in intracellular Ca^2+^ concentration (Bernstein et al., 1998). Responses exhibited a significant reduction in AUC at DIV 21 (54.38 ± 2.56, *n* = 3) and DIV 28 (62.08 ± 4.51, *n* = 3), compared to DIV 7 (70.76 ± 5.11, *n* = 3), revealing a change in the total amount of calcium trafficked in and out of the cell, as the cultures develop (Figure 7A). Calcium clearance, measured via DT, is significantly faster at DIV 21 (65.42 ± 13.56s, *n* = 3), compared to DIV 7 (82.95 ± 11.16s, *n* = 3) and DIV 14 (80.66 ± 22.21s, *n* = 3) (Figure 7B). Furthermore, there was a significant (*p* < 0.05) increase in RT of responses between DIV 7 (3.75s ± 0.202s, *n* = 3) and DIV 28 (6.04s ± 0.56s, *n* = 3) (Figure 7C). Latency remained stable at approximately 23 s throughout (Figure 7D).

The P2Y_1_R, plays a prominent role in the rise time component in Ca^2+^ kinetics (James and Butt, 2001). We observed significant increases in the rise time of the Ca^2+^ transients, in the generated astrocytes, at later developmental stages (DIV 21 and 28, Figure 7C). These findings may indicate an increase in the number of P2Y and IP_3_ receptors in these cells, which enable the release of further Ca^2+^ from the ER. Alternatively, the increase in RT may be attributed to an evolution of the P2Y receptor ability to respond to ATP, at late developmental stages (Hamilton et al., 2008).

### P2Y_1_R Is the Major Contributor to Calcium Transients in Mouse Embryonic Stem Cell Line-Derived Astrocytes

**FIGURE 8:**
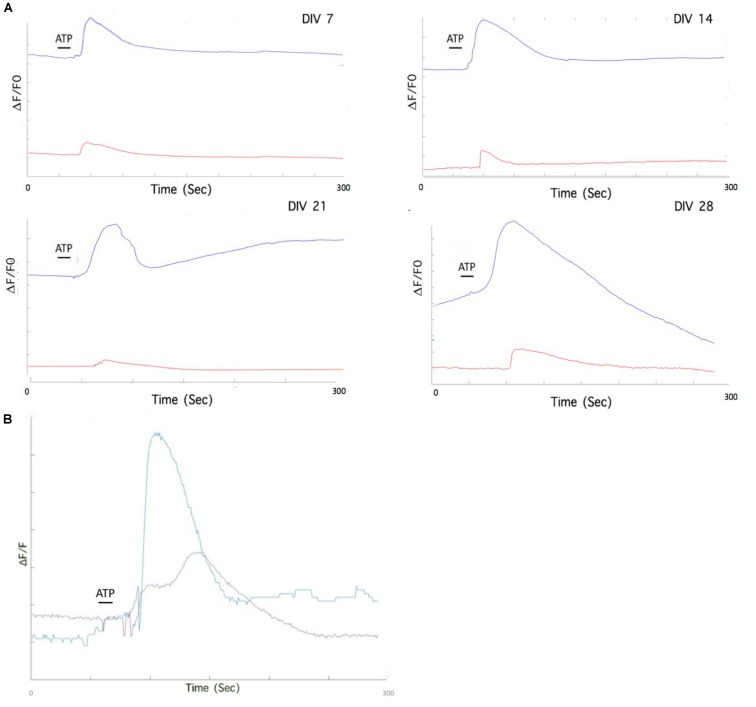
Pharmacological treatment of mESC derived astrocytes isolates individual purinoceptor contributions to Ca^2+^ elevation. **(A)** Individual astrocytes from MRS2179 treated DIV 7-DIV 28 cultures exhibit stunted Ca^2+^ responses after ATP application (red traces), compared to untreated astrocytes (blue traces). **(B)** Pharmacological treatment of mESC derived astrocytes with phospholipase C (PLC) inhibitor, U73122. Bath incubation of DIV 28 astrocytes with U73122 results in a stunted response upon ATP stimulation. Purple trace. ATP response after U73122 treatment; Blue trace, ATP response without U73122 treatment.

We used a P2Y_1_R selective antagonist (MRS2179) to offer further evidence of the contribution of the metabotropic pathway to Ca^2+^ transients. Preliminary data indicates that blocking of the metabotropic receptor P2Y_1_R has distinct effects on astrocytic responses to ATP in DIV 7 to DIV 28 cultures (Figure 8A). We also inhibited PLC, which hydrolyzes the membrane phospholipid PIP2 to form IP_3_. After the application of U73122, ATP stimulation resulted in a stunted Ca^2+^ response (Figure 8B).

## Discussion

Rapid progress has been made in converting stem cells into other types of neural cells, such as neurons, Schwann cells and oligodendrocytes (Ban et al., 2007; Zhang K. et al., 2016). However, inducting astrocytes from stem cells remains largely an underdeveloped area and the majority of current protocols are time consuming, lack granularity in astrocytic subtype generation and often are not as efficient as neural induction methods. In this study, we differentiated mouse embryonic stem cells to produce functional astrocytes via the combination of two altered protocols (Peljto et al., 2010; Kuegler et al., 2012). Our method produces migrating astrocytes after only 13 days *in vitro* (including EB formation and seeding). Most established protocols require more than 80 days to generate astrocytes either from mouse (Roybon et al., 2013) or human (Hu et al., 2010) ESCs, while only recently astrocytes were differentiated from human induced pluripotent stem cells in 30 days (Tcw et al., 2017). Our astrocyte induction method is significantly accelerated, compared to all these techniques. In addition, it does not require sorting processes, or inflammatory stimulants (Shaltouki et al., 2013; Tcw et al., 2017) and therefore is simpler and easier to implement.

Developmental studies have shown that the sequence of marker appearance during the initial, embryonic phase of gliogenesis is ALDH1L1 > S100β > GFAP (Roybon et al., 2013). This is supported by our data, as at DIV 7 expression of ALDH1L1 is highest, followed by S100β and GFAP. We also observed, a significant reduction in expression of S100β from DIV 7 to DIV14, with a subsequent bounce at DIV 21. We hypothesize that this expression pattern might be linked to the developmental repression of the S100β protein, during differentiation of NPC and the reappearance of the marker in mature astrocytes (Patro et al., 2015).

The glutamate-aspartate transporter (GLAST or EAAT1) is a membrane bound protein involved in the uptake of glutamate from the synaptic cleft. It is expressed in both developing and mature astrocytes and it is most prominent in the cerebellum, hippocampus and forebrain (Kugler and Schleyer, 2004; Kim et al., 2011). In our cultures, numerous GFAP^+^ astrocytes expressed GLAST (EAAT1) particularly on their membrane, where this cotransporter is bound (Figure 5C; Ryan et al., 2010; Pajarillo et al., 2019). However, we did observe GLAST^+^ cells that were negative for GFAP (Figure 5D), particularly in earlier DIV. Similarly, co-stained cultures for GFAP, S100β and ALDH1L1, revealed heterogeneous expression patterns, within and across different developmental time-points. We identified GFAP^–^ cells that were ALDH1L1^+^/S100β^+^ (Figures 4A,B, white arrows) and GFAP^+^ cells that were ALDH1L1^+^/S100β^+^ (Figures 4A,B, yellow arrows).

These findings align with *in vitro* studies that highlight heterogeneous populations of astrocytes in neonatal primary cell cultures (Tetsuya et al., 2006), in differentiating stem cell cultures (Kuegler et al., 2012) and investigations that have identified astrocytes with diverse inter- and intra-regional characteristics within the mammalian CNS (Ben Haim and Rowitch, 2017). We hypothesize that the derived astrocytes in our protocol are at different stages of maturation, rather than differentiating toward distinct developmental paths. This would also explain the sparse co-labeling of GFAP^+^ and ALDH1L^+^ cells in our cultures, as well as the predominance of GFAP^+^/GLAST^+^ astrocytes at later developmental stages (DIV 21/28). These astrocytes should be able to recycle glutamate in neuron-astrocyte co-cultures, enabling further investigations in this area.

Ca^2+^ release and uptake in astrocytes is regulated by multiple pathways and receptors. Examples of include the P2X_1__–__5_ (Pankratov et al., 2009), NMDA (Palygin et al., 2010), cannabinoid receptor CB1 (Rasooli-Nejad et al., 2014) and a1-noradrenaline receptors (Pankratov and Lalo, 2015). In this study we focused on the purinergic receptors and in particular the P2Y_1_R. The purinergic signaling system is one of the main extracellular signaling systems that integrates neuronal-glial and glial–glial circuits in the nervous system (Pascual et al., 2005). ATP is the principal purinergic signaler and is released from cells by several mechanisms, such as exocytosis, diffusion through membrane channels and transporters mediated release. Purinergic receptors initiate numerous downstream signaling cascades, which mediate physiological and pathological astrocyte responses (Franke et al., 2012). We observed significant expression of P2Y_1_R purinoceptors in GFAP^+^ and GFAP^–^ cells (Figure 5A). Having established receptor presence, we also assessed purinergic responses in the derived astrocytes, as an initial step in verifying their potential functionality, within a cultured neural network.

Application of ATP to cultures resulted in a rapid and transient rise in intracellular Ca^2+^ followed by an exponential decay to baseline. The majority of the responses were monophasic, although we did observe some biphasic responses as well (containing a prolonged component during the decay phase), particularly in the later developmental time-points (Hashioka et al., 2014). Biphasic responses may be due to late influx of Ca^2+^ from store operated channels (SOC). Calcium clearance (Figure 7B) was significantly faster at later DIV, which might be linked to the developmental stage of the astrocytes. Variability in astrocyte Ca^2+^ responses to ATP stimulation may also be linked to the diverse protein expression patterns that we observed in the double immunostained cultures and may be related to the maturation of these astrocytes.

Analysis of receptor specific pathways was probed by using receptor specific inhibitors. P2Y_1_R is the main purinergic receptor involved in transmission of Ca^2+^ waves between astrocytes (Fam et al., 2000). When our astrocytes were treated with MRS2179 (a P2Y_1_R antagonist) they exhibited significantly reduced Ca^2+^ responses to ATP stimulation (Figure 8A). Similar effects of P2Y_1_R inhibition by MRS2179 have been reported *in vivo* studies of white matter astrocytes (Hamilton et al., 2008). Furthermore, inhibition of the PLC pathway resulted in a stunted Ca^2+^ response to ATP, signifying the involvement of IP_3_ in Ca^2+^ elevation in our astrocytes. Our experiments provide preliminary evidence that Ca^2+^ elevation in the generated astrocytes could be a result of the metabotropic pathway, and possibly initiated at the P2Y_1_R receptor. Further experiments with selective agonists and antagonists will isolate the specific P2Y receptor involved.

## Conclusion

In this study, we derived functional astrocytes from Mouse Embryonic Stem Cell Line in under 2 weeks. Currently, this is the fastest protocol available to produce astrocytes. The generated astrocytes respond to ATP (50 μM) stimulation via intracellular Ca^2+^ concentration elevation and express proteins associated to astrocytic development and maturation. Differential transient responses to ATP and diverse expression profiles for ALDH1L1, S100β, GLAST, and GFAP reveal a heterogeneous astrocyte population, at different stages of development (DIV 7-28). Furthermore, we provide preliminary evidence that the metabotropic purinoceptor P2Y_1_R may be the main factor in ATP induced Ca^2+^ activity. Due to its simplicity, reproducibility and speed, we believe our protocol will be useful in many future investigations, concerning the involvement of astrocytes in neuronal synaptic development and support, and the role of purinergic signaling in activity propagation *in vitro* neural networks.

## Data Availability Statement

The datasets generated for this study are available on request to the corresponding author.

## Author Contributions

DJ: collection and assembly of data, data analysis and interpretation, and manuscript writing. SN: financial support, data analysis and interpretation, and manuscript writing. ED: conception and design, financial support, data analysis and interpretation, manuscript writing, and final approval of manuscript.

## Conflict of Interest

The authors declare that the research was conducted in the absence of any commercial or financial relationships that could be construed as a potential conflict of interest.

## Supplementary Material

FIGURE S1 **(A)** Nanog expression levels relative to GAPDH reveal statistically significant downregulation of the gene by DIV 28. **(B)** Representative culture example at DIV 7 with double immunofluorescent staining with astrocytic marker GFAP (green) and neuronal precursor marker Nestin (red). Under our astrocyte induction protocol, no Nestin was present at DIV 7 or later, indicating the absence of neurons and their precursors. Scale bar 20μm. ^∗^*P* < 0.05.

## References

[B1] Adams K. L. Gallo V. (2018). The diversity and disparity of the glial scar. *Nat. Neurosci.* 21 9–15. 10.1038/s41593-017-0033-9 29269757 PMC5937232

[B2] Adler J. Parmryd I. (2010). Quantifying colocalization by correlation: the pearson correlation coefficient is superior to the Mander’s overlap coefficient. *Cytom. Part A* 77 733–742. 10.1002/cyto.a.20896 20653013

[B3] Ban J. Bonifazi P. Pinato G. Broccard F. D. Studer L. Torre V. (2007). Embryonic stem cell-derived neurons form functional networks in vitro. *Stem Cells* 25 738–749. 10.1634/stemcells.2006-0246 17110621

[B4] Bazargani N. Attwell D. (2016). Astrocyte calcium signaling: the third wave. *Nat. Neurosci.* 19 182–189. 10.1038/nn.4201 26814587

[B5] Ben Haim L. Rowitch D. (2017). Functional diversity of astrocytes in neural circuit regulation. *Nat. Rev. Neurosci.* 18 31–41. 10.1038/nrn.2016.159 27904142

[B6] Bernstein M. Behnisch T. Balschun D. Reymann K. G. Reiser G. (1998). Pharmacological characterisation of metabotropic glutamatergic and purinergic receptors linked to Ca2+ signalling in hippocampal astrocytes. *Neuropharmacology* 37 169–178. 10.1016/s0028-3908(98)00012-4 9680241

[B7] Cahoy J. D. Emery B. Kaushal A. Foo L. C. Zamanian J. L. Christopherson K. S. (2008). A transcriptome database for astrocytes, neurons, and oligodendrocytes: a new resource for understanding brain development and function. *J. Neurosci.* 28 264–278. 10.1523/jneurosci.4178-07.2008 18171944 PMC6671143

[B8] Clarke L. E. Barres B. A. (2013). Emerging roles of astrocytes in neural circuit development. *Nat. Rev. Neurosci.* 14:311. 10.1038/nrn3484 23595014 PMC4431630

[B9] Cragnolini A. B. Montenegro G. Friedman W. J. Mascó D. H. (2018). Brain-region specific responses of astrocytes to an in vitro injury and neurotrophins. *Mol. Cell. Neurosci.* 88 240–248. 10.1016/j.mcn.2018.02.007 29444457

[B10] Delivopoulos E. Murray A. F. (2011). Controlled adhesion and growth of long term glial and neuronal cultures on parylene-C. *PLoS One* 6:e25411. 10.1371/journal.pone.0025411 21966523 PMC3178637

[B11] Delivopoulos E. Murray A. F. MacLeod N. K. Curtis J. C. (2009). Guided growth of neurons and glia using microfabricated patterns of parylene-C on a SiO2 background. *Biomaterials* 30 2048–2058. 10.1016/j.biomaterials.2008.12.049 19138795

[B12] Emdad L. D’Souza S. L. Kothari H. P. Qadeer Z. A. Germano I. M. (2011). Efficient differentiation of human embryonic and induced pluripotent stem cells into functional astrocytes. *Stem Cells Dev.* 21 404–410. 10.1089/scd.2010.0560 21631388

[B13] Eng L. F. Ghirnikar R. S. Lee Y. L. (2000). Glial fibrillary acidic protein: GFAP-thirty-one years (1969-2000). *Neurochem. Res.* 25 1439–1451. 11059815 10.1023/a:1007677003387

[B14] Fam S. R. Gallagher C. J. Salter M. W. (2000). P2Y1 purinoceptor-mediated Ca2+ signaling and Ca2+ wave propagation in dorsal spinal cord astrocytes. *J. Neurosci.* 20 2800–2808. 10.1523/jneurosci.20-08-02800.2000 10751431 PMC6772222

[B15] Franke H. Verkhratsky A. Burnstock G. Illes P. (2012). Pathophysiology of astroglial purinergic signalling. *Purinergic Signal* 8 629–657. 10.1007/s11302-012-9300-0 22544529 PMC3360100

[B16] Gee K. R. Brown K. A. Chen W. N. Bishop-Stewart J. Gray D. Johnson I. (2000). Chemical and physiological characterization of fluo-4 Ca(2+)-indicator dyes. *Cell Calcium* 27 97–106. 10.1054/ceca.1999.0095 10756976

[B17] Hamilton N. Vayro S. Kirchhoff F. Verkhratsky A. Robbins J. Gorecki D. C. (2008). Mechanisms of ATP- and glutamate-mediated calcium signaling in white matter astrocytes. *Glia* 56 734–749. 10.1002/glia.20649 18293404

[B18] Han X. Chen M. Wang F. Windrem M. Wang S. Shanz S. (2013). Forebrain engraftment by human glial progenitor cells enhances synaptic plasticity and learning in adult mice. *Cell Stem Cell* 12 342–353. 10.1016/j.stem.2012.12.015 23472873 PMC3700554

[B19] Hashioka S. Wang Y. F. Little J. P. Choi H. B. Klegeris A. McGeer P. L. (2014). Purinergic responses of calcium-dependent signaling pathways in cultured adult human astrocytes. *BMC Neurosci.* 15:18. 10.1186/1471-2202-15-18 24447580 PMC3903030

[B20] Hu B.-Y. Weick J. P. Yu J. Ma L.-X. Zhang X.-Q. Thomson J. A. (2010). Neural differentiation of human induced pluripotent stem cells follows developmental principles but with variable potency. *Proc. Natl. Acad. Sci. U.S.A.* 107 4335–4340. 10.1073/pnas.0910012107 20160098 PMC2840097

[B21] Itoh N. Itoh Y. Tassoni A. Ren E. Kaito M. Ohno A. (2018). Cell-specific and region-specific transcriptomics in the multiple sclerosis model: focus on astrocytes. *Proc. Natl. Acad. Sci. U.S.A.* 115 E302–E309. 10.1073/pnas.1716032115 29279367 PMC5777065

[B22] James G. Butt A. M. (2001). P2X and P2Y purinoreceptors mediate ATP-evoked calcium signalling in optic nerve glia in situ. *Cell Calcium* 30 251–259. 10.1054/ceca.2001.0232 11587549

[B23] James L. R. Andrews S. Walker S. de Sousa P. R. S. Ray A. Russell N. A. (2011). High-throughput analysis of calcium signalling kinetics in astrocytes stimulated with different neurotransmitters. *PLoS One* 6:e26889. 10.1371/journal.pone.0026889 22046396 PMC3201978

[B24] Kang M. Othmer H. G. (2009). Spatiotemporal characteristics of calcium dynamics in astrocytes. *Chaos* 19 1–21. 10.1063/1.3206698 PMC2852438 19792041

[B25] Kim K. Lee S.-G. Kegelman T. P. Su Z.-Z. Das S. K. Dash R. (2011). Role of Excitatory Amino Acid Transporter-2 (EAAT2) and glutamate in neurodegeneration: opportunities for developing novel therapeutics. *J. Cell. Physiol.* 226 2484–2493. 10.1002/jcp.22609 21792905 PMC3130100

[B26] Kleiderman S. Sá J. V. Teixeira A. P. Brito C. Gutbier S. Evje L. G. (2016). Functional and phenotypic differences of pure populations of stem cell-derived astrocytes and neuronal precursor cells. *Glia* 64 695–715. 10.1002/glia.22954 26689134

[B27] Krencik R. Weick J. P. Liu Y. Zhang Z.-J. Zhang S.-C. (2011). Specification of transplantable astroglial subtypes from human pluripotent stem cells. *Nat. Biotechnol.* 29 528–534. 10.1038/nbt.1877 21602806 PMC3111840

[B28] Krencik R. Zhang S.-C. (2011). Directed differentiation of functional astroglial subtypes from human pluripotent stem cells. *Nat. Protoc.* 6:1710. 10.1038/nprot.2011.405 22011653 PMC3198813

[B29] Kuegler P. B. Baumann B. A. Zimmer B. Keller S. Marx A. Kadereit S. (2012). GFAP-independent inflammatory competence and trophic functions of astrocytes generated from murine embryonic stem cells. *Glia* 60 218–228. 10.1002/glia.21257 22072312

[B30] Kugler P. Schleyer V. (2004). Developmental expression of glutamate transporters and glutamate dehydrogenase in astrocytes of the postnatal rat hippocampus. *Hippocampus* 14 975–985. 10.1002/hipo.20015 15390174

[B31] Lee H. J. Suk J. E. Patrick C. Bae E. J. Cho J. H. Rho S. (2010). Direct transfer of α-synuclein from neuron to astroglia causes inflammatory responses in synucleinopathies. *J. Biol. Chem.* 285 9262–9272. 10.1074/jbc.M109.081125 20071342 PMC2838344

[B32] Lent R. Azevedo F. A. C. Andrade-Moraes C. H. Pinto A. V. O. (2012). How many neurons do you have? Some dogmas of quantitative neuroscience under revision. *Eur. J. Neurosci.* 35 1–9. 10.1111/j.1460-9568.2011.07923.x 22151227

[B33] Moeton M. Stassen O. M. J. A. Sluijs J. A. van der Meer V. W. N. Kluivers L. J. van Hoorn H. (2016). GFAP isoforms control intermediate filament network dynamics, cell morphology, and focal adhesions. *Cell. Mol. Life Sci.* 73 4101–4120. 10.1007/s00018-016-2239-5 27141937 PMC5043008

[B34] Molofsky A. V. Deneen B. (2015). Astrocyte development: a guide for the perplexed. *Glia* 63 1320–1329. 10.1002/glia.22836 25963996

[B35] Nagayasu T. Miyata S. Hayashi N. Takano R. Kariya Y. Kamei K. (2005). Heparin structures in FGF-2-dependent morphological transformation of astrocytes. *J. Biomed. Mater. Res. - Part A* 74 374–380. 10.1002/jbm.a.30338 15973728

[B36] Neary J. T. Kang Y. Bu Y. Yu E. Akong K. Peters C. M. (1999). Mitogenic signaling by ATP/P2Y purinergic receptors in astrocytes: involvement of a calcium-independent protein kinase C, extracellular signal-regulated protein kinase pathway distinct from the phosphatidylinositol-specific phospholipase C/calcium pathway. *J. Neurosci.* 19 4211–4220. 10.1523/jneurosci.19-11-04211.1999 10341225 PMC6782585

[B37] Okada Y. Sasaki T. Oku Y. Takahashi N. Seki M. Ujita S. (2012). Preinspiratory calcium rise in putative pre-Botzinger complex astrocytes. *J. Physiol.* 590 4933–4944. 10.1113/jphysiol.2012.231464 22777672 PMC3487046

[B38] Otsu Y. Couchman K. Lyons D. G. Collot M. Agarwal A. Mallet J. M. (2015). Calcium dynamics in astrocyte processes during neurovascular coupling. *Nat. Neurosci.* 18 210–218. 10.1038/nn.3906 25531572 PMC4651918

[B39] Pajarillo E. Rizor A. Lee J. Aschner M. Lee E. (2019). The role of astrocytic glutamate transporters GLT-1 and GLAST in neurological disorders: potential targets for neurotherapeutics. *Neuropharmacology* 161:107559. 10.1016/j.neuropharm.2019.03.002 30851309 PMC6731169

[B40] Palygin O. Lalo U. Verkhratsky A. Pankratov Y. (2010). Ionotropic NMDA and P2X1/5 receptors mediate synaptically induced Ca2+ signalling in cortical astrocytes. *Cell Calcium* 48 225–231. 10.1016/j.ceca.2010.09.004 20926134

[B41] Pankratov Y. Lalo U. (2015). Role for astroglial α1-adrenoreceptors in gliotransmission and control of synaptic plasticity in the neocortex. *Front. Cell. Neurosci.* 9 230. 10.3389/fncel.2015.00230 26136663 PMC4468378

[B42] Pankratov Y. Lalo U. Krishtal O. A. Verkhratsky A. (2009). P2X receptors and synaptic plasticity. *Neuroscience* 158 137–148. 10.1016/j.neuroscience.2008.03.076 18495357

[B43] Pascual O. Casper K. B. Kubera C. Zhang J. Revilla-Sanchez R. Sul J.-Y. (2005). Astrocytic purinergic signaling coordinates synaptic networks. *Science* 310 113–116. 10.1126/science.1116916 16210541

[B44] Patro N. Naik A. Patro I. K. (2015). Differential temporal expression of S100β in developing rat brain. *Front. Cell. Neurosci.* 9:87. 10.3389/fncel.2015.00087 25852479 PMC4364248

[B45] Peljto M. Dasen J. S. Mazzoni E. O. Jessell T. M. Wichterle H. (2010). Functional diversity of ESC-derived motor neuron subtypes revealed through intraspinal transplantation. *Cell Stem Cell* 7 355–366. 10.1016/j.stem.2010.07.013 20804971 PMC2933095

[B46] Raos B. J. Simpson M. C. Doyle C. S. Murray A. F. Graham E. S. Unsworth C. P. (2018). Patterning of functional human astrocytes onto parylene-C/SiO_2_ substrates for the study of Ca^2+^ dynamics in astrocytic networks. *J. Neural Eng.* 15:036015. 10.1088/1741-2552/aaae1d 29424361

[B47] Raponi E. Agenes F. Delphin C. Assard N. Baudier J. Legraverend C. (2007). S100B expression defines a state in which GFAP-expressing cells lose their neural stem cell potential and acquire a more mature developmental stage. *Glia* 55 165–177. 10.1002/glia.20445 17078026 PMC2739421

[B48] Rasooli-Nejad S. Palygin O. Lalo U. Pankratov Y. (2014). Cannabinoid receptors contribute to astroglial Ca^2+^-signalling and control of synaptic plasticity in the neocortex. *Philos. Trans. R. Soc. Lond. B. Biol. Sci.* 369:20140077. 10.1098/rstb.2014.0077 25225106 PMC4173298

[B49] Robertson P. L. Goldstein G. W. (1988). Heparin inhibits the growth of astrocytes in vitro. *Brain Res.* 447 341–345. 10.1016/0006-8993(88)91137-7 3390702

[B50] Roybon L. Lamas N. Garcia-Diaz A. Yang E. Sattler R. Jackson-Lewis V. (2013). Human stem cell-derived spinal cord astrocytes with defined mature or reactive phenotypes. *Cell Rep.* 4 1035–1048. 10.1016/j.celrep.2013.06.021 23994478 PMC4229657

[B51] Ryan R. M. Kortt N. C. Sirivanta T. Vandenberg R. J. (2010). The position of an arginine residue influences substrate affinity and K+ coupling in the human glutamate transporter. *EAAT1 J. Neurochem.* 114 565–575. 10.1111/j.1471-4159.2010.06796.x 20477940

[B52] Santos R. Vadodaria K. C. Jaeger B. N. Mei A. Lefcochilos-Fogelquist S. Mendes A. P. D. (2017). Differentiation of inflammation-responsive astrocytes from glial progenitors generated from human induced pluripotent stem cells. *Stem Cell Reports* 8 1757–1769. 10.1016/j.stemcr.2017.05.011 28591655 PMC5470172

[B53] Selinfreund R. H. Barger S. W. Pledger W. J. Van Eldik L. J. (1991). Neurotrophic protein S100β stimulates glial cell proliferation. *Proc. Natl. Acad. Sci. U.S.A.* 88 3554–3558. 10.1073/pnas.88.9.3554 1902567 PMC51490

[B54] Shaltouki A. Peng J. Liu Q. Rao M. S. Zeng X. (2013). Efficient generation of astrocytes from human pluripotent stem cells in defined conditions. *Stem Cells* 31 941–952. 10.1002/stem.1334 23341249

[B55] Shigetomi E. Patel S. Khakh B. S. (2016). Probing the complexities of astrocyte calcium signaling. *Trends Cell Biol.* 26 300–312. 10.1016/j.tcb.2016.01.003 26896246 PMC4946798

[B56] Tcw J. Wang M. Pimenova A. A. Bowles K. R. Hartley B. J. Lacin E. (2017). An efficient platform for astrocyte differentiation from human induced pluripotent stem cells. *Stem Cell Rep.* 9 600–614. 10.1016/j.stemcr.2017.06.018 28757165 PMC5550034

[B57] Tetsuya I. Ichiro N. I. Korblum H. Sofroniew V. M. (2006). Phenotypic and functional heterogeneity of GFAP-expressing cells in vitro: differential expression of LeX/CD15 by GFAP-expressing multipotent neural stem cells and non-neurogenic astrocytes. *Glia* 53 277–293. 10.1002/glia.20281 16267834

[B58] Tian E. Sun G. Sun G. Chao J. Ye P. Warden C. (2016). Small-molecule-based lineage reprogramming creates functional astrocytes. *Cell Rep.* 16 781–792. 10.1016/j.celrep.2016.06.042 27396343 PMC9228989

[B59] Tiwari N. Pataskar A. Péron S. Thakurela S. Sahu S. K. Figueres-Oñate M. (2018). Stage-specific transcription factors drive astrogliogenesis by remodeling gene regulatory landscapes. *Cell Stem Cell* 23 557.e–571.e. 10.1016/j.stem.2018.09.008 30290178 PMC6179960

[B60] Tong X. Ao Y. Faas G. Nwaobi S. Olsen M. Sofroniew M. V. (2016). Astrocyte Kir4.1 ion channel deficits contribute to neuronal dysfunction in Huntington’s disease model mice Xiaoping. *Nat. Neurosci.* 17 1–21. 10.1038/nn.3691 PMC4064471 24686787

[B61] Unsworth C. P. Holloway H. Delivopoulos E. Murray A. F. Simpson M. C. Dickinson M. E. (2011). Patterning and detailed study of human hNT astrocytes on parylene-C/silicon dioxide substrates to the single cell level. *Biomaterials* 32 6541–6550. 10.1016/j.biomaterials.2011.05.041 21641029

[B62] Vala M. H. J. Baxi A. (2013). A review on Otsu image segmentation algorithm. *Int. J. Adv. Res. Comput. Eng. Technol.* 2 387–389. 10.1007/s11548-009-0389-8 20033505

[B63] van Bodegraven E. J. van Asperen J. V. Robe P. A. J. Hol E. M. (2019). Importance of GFAP isoform-specific analyses in astrocytoma. *Glia* 67 1417–1433. 10.1002/glia.23594 30667110 PMC6617972

[B64] Zhang K. Chen C. Yang Z. He W. Liao X. Ma Q. (2016). sensory response of transplanted astrocytes in adult mammalian cortex in vivo. *Cereb. Cortex* 26 3690–3704. 10.1093/cercor/bhw213 27405333 PMC5004757

[B65] Zhang L. Yin J.-C. Yeh H. Ma N.-X. Lee G. Chen X. A. (2015). Small molecules efficiently reprogram human astroglial cells into functional neurons. *Cell Stem Cell* 17 735–747. 10.1016/j.stem.2015.09.012 26481520 PMC4675726

[B66] Zhang Y. Sloan S. A. Clarke L. E. Caneda C. Plaza C. A. Blumenthal P. D. (2016). Purification and characterization of progenitor and mature human astrocytes reveals transcriptional and functional differences with mouse. *Neuron* 89 37–53. 10.1016/j.neuron.2015.11.013 26687838 PMC4707064

[B67] Zhou S. Szczesna K. Ochalek A. Kobolák J. Varga E. Nemes C. (2016). Neurosphere based differentiation of human IPSC improves astrocyte differentiation. *Stem Cells Int.* 2016:4937689. 10.1155/2016/4937689 26798357 PMC4699090

