# Supplementary material for: Fast and Efficient Differentiation of Mouse Embryonic Stem Cells Into ATP-Responsive Astrocytes
Source: Front Cell Neurosci. 2020 Jan 21;13:579. doi: 10.3389/fncel.2019.00579 (PMC6985097; doi:10.3389/fncel.2019.00579)
Supplement: FIGURE S1 — (A) Nanog expression levels relative to GAPDH reveal statistically significant downregulation of the gene by DIV 28. (B) Representative culture example at DIV 7 with double immunofluorescent staining with astrocytic marker GFAP (green) and neuronal precursor marker Nestin (red). Under our astrocyte induction protocol, no Nestin was present at DIV 7 or later, indicating the absence of neurons and their precursors. Scale bar 20μm. ∗P < 0.05. [file Data_Sheet_1.docx]

**Supplementary Information**

**Reverse Transcription Polymerase Chain Reaction**

Total RNA from 3 separate culture experiments was isolated using the QIAGEN Rneasy Kit according to the manufacturer’s instructions, measured in a spectrophotometer and purity ensured by 260/280 nm ratio of greater than 1.95 for all samples. Each RNA sample was treated with Dnase I from the Rneasy Kit, to eliminate any genomic contamination. The QIAGEN QuantiTect Reverse Transcription Kit was used to transcribe 1 μg of RNA to cDNA. PCR was conducted with the DreamTaq Green PCR Master Mix (ThermoFisher). The protocol consisted of an initial denaturation step at 95°C for 2 min and 35 cycles of: i) denaturation step 30 s at 95°C, ii) annealing step 30 s at 55°C and iii) extension step 1 min at 72°C. A final extension step of 5 min at 72°C was conducted at the end. Separation of PCR products was performed in a 1% agarose gel via electrophoresis and visualization was done with SYBR safe DNA gel stain (ThermoFisher). Digital photographs of the gels were taken with the Syngene U Genius 3 System (SLS) and cDNA levels relative to the housekeeping gene Gapdh were calculated using PCR band densitometry (ImageJ NIH – National Institutes of Health). Primers used were *Nanog:* 5’-CAGAAGGGCTCAGCACCAGTG-3’ (forward) and 5’-CAAGTTGGGTTGGTCCAGGTCTG-3’ (reverse), *Iba1:* 5'-AGCCTGAGGAGATTTCAACAGAA-3' (forward) and 5'-TTTGGACGGCAGATCCTCAT-3' (reverse).

Table 1. Concentrations and brands of primary and secondary antibodies

|  | Description | Concentration | Source |
| --- | --- | --- | --- |
| **Primary** | | | |
| GFAP | Chicken polyclonal | 1:500 | Aves |
| GFAP | Rabbit polyclonal | 1:250 | Abcam |
| ALDH1L1 | Rabbit polyclonal | 1:100 | Abcam |
| S100β | Rabbit polyclonal | 1:250 | Abcam |
| P2Y_1_R | Rabbit polyclonal | 1:250 | Alomone |
| GLAST | Rabbit polyclonal | 1:250 | Abcam |
| Nestin | Rat monoclonal | 1:500 | Santa Cruz |
| **Secondary** | | | |
| Anti-rabbit | 488 polyclonal | 1:500 | Alexa Fluor |
| Anti-chicken | 594 polyclonal | 1:500 | Alexa Fluor |
| Anti-rat | 594 polyclonal | 1:250 | Alexa Fluor |
| Anti-mouse | 594 polyclonal | 1:500 | Alexa Fluor |


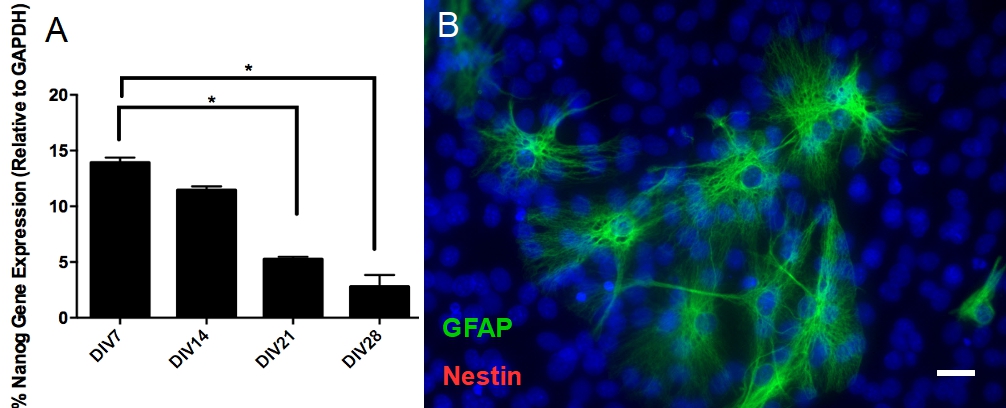


FIGURE S1 | (A) Nanog expression levels relative to GAPDH reveal statistically significant downregulation of the gene by DIV 28. (B) Representative culture example at DIV 7 with double immunofluorescent staining with astrocytic marker GFAP (green) and neuronal precursor marker Nestin (red). Under our astrocyte induction protocol, no Nestin was present at DIV 7 or later, indicating the absence of neurons and their precursors. Scale bar 20μm, *P < 0.05.
